# Effects of acupuncture treatment on posttraumatic headache after traumatic brain injury in patients: A protocol for systematic review

**DOI:** 10.1097/MD.0000000000029158

**Published:** 2022-05-13

**Authors:** Xi Wen, Yang Yang, Yunhai Li, Tong Liu, Yue Liu, Xiaoyin Wang, Fangyi Lu, Chanzhen Yu, Nenggui Xu

**Affiliations:** a Guangzhou University of Chinese Medicine, Guangzhou, China; b Guangdong Second Traditional Chinese Medicine Hospital, Guangzhou, China; c The Fifth Clinical Medical School of Guangzhou University of Chinese Medicine, Guangzhou, China; d Affiliated Jiangmen Traditional Chinese Medicine Hospital of Jinan University, Jinan University, Guangzhou, China; e The Third Affiliated Hospital of Guangzhou University of Chinese Medicine, Guangzhou, China.

**Keywords:** acupuncture, posttraumatic headache, systematic review, traumatic brain injury

## Abstract

**Background:**

Posttraumatic headache (PTH) after traumatic brain injury (TBI) is a common clinical symptom, which refers to a headache that occurs after TBI. Acupuncture is often used for the treatment of such patients in China, and significant clinical effects have been achieved. However, to date, its efficacy has not been methodically evaluated. The purpose of this systematic review is to provide evidence to prove the effectiveness of acupuncture in the treatment of PTH in patients with TBI.

**Methods:**

This systematic review will be conducted in accordance with the preferred reporting items for systematic review and meta-analysis protocols. The following electronic databases will be searched from their inception to February 2022: PubMed, Web of Science, Embase, PsycINFO, the Cochrane Library, and Chinese databases such as Chinese Biomedical Literature (CBM), Chinese Medical Current Content (CMCC), Chinese Scientific Journal Database (VIP), WanFang Database, and China National Knowledge Infrastructure (CNKI). No language restrictions will be applied to the search strategy. Randomized controlled trials and cohort and case-control studies that met the inclusion and exclusion criteria will be included in this study. The meta-analysis will be performed using RevMan 5.3 software. Each session of this systematic review will be conducted independently by 2 members.

**Results:**

This review evaluates the efficacy of acupuncture in the treatment of PTH after TBI.

**Conclusion:**

This review provides substantial evidence for the clinical application of acupuncture in PTH treatment after TBI.

**Ethics and dissemination:**

Since the data in this study will be retrieved from published trials, therefore the Patient Consent Statement and Ethical Approval are not required. We will disseminate our results by publishing the research in a peer-reviewed journal.

**Trail registration number:**

The protocol was registered in INPLASY (INPLASY 202220073).

## 1 Introduction

Posttraumatic headache (PTH) after traumatic brain injury (TBI) is a common clinical symptom that refers to a headache that occurs after TBI.^[1]^ In recent years, with the increase in traffic accidents, work-related injuries, sports injuries, and other accidents in my country, the incidence of TBI has been increasing every year. A study has shown that the annual incidence of TBI is approximately 1000 to 2000 per 1 million people, with an annual growth rate of 4.67% in China.^[2]^ It can be seen that the incidence and prevalence of TBI in my country are generally increasing rapidly.

PTH is one of the most common symptoms after TBI and often causes great suffering in patients.^[3]^ Effective treatment with PTH can reduce pain in patients and is also conducive to TBI treatment. On the contrary, if TBI cannot be treated in a timely and effective manner, it will lead to repeated episodes of the disease and cause serious distress to patients.^[4]^ Therefore, ensuring the effectiveness of PTH treatment is currently a hot research topic. There have been many studies on the efficacy of acupuncture in the treatment of TBI in China, and many studies have confirmed that acupuncture has a better effect than conventional Western medicine.^[5]^ To date, no systematic review or meta-analysis has been conducted regarding the effectiveness of acupuncture in the treatment of PTH after TBI. Therefore, there is an urgent need to provide high-quality evidence for the application of acupuncture in the management of PTH after TBI.

## 2 Methods

This systematic review was prospectively registered on the INPLASY system and has already achieved register number INPLASY202220073. This protocol was strictly developed according to the Preferred Guidelines for Systematic Reviews (PRISMA-P). The study will begin in March 2022 and is expected to be completed by September 2022.

### 2.1 Inclusion and exclusion criteria

#### 2.1.1 Types of studies

Randomized controlled trials and cohort and case-control studies that met the inclusion and exclusion criteria will be included in this study.

#### 2.1.2 Types of participants

Adults over the age of 18 years with headache after TBI and meeting the criteria for a diagnosis of PTH will be included, regardless of sex, race, nationality, and medical institution.

#### 2.1.3 Types of interventions

All patients who had used acupuncture or related technical interventions, such as acupuncture, electro-acupuncture, moxibustion, acupressure, acupoint injection, cupping, laser acupuncture, ear acupuncture, scalp acupuncture, acupoint bloodletting therapy, fire acupuncture, intradermal acupuncture, and acupoint catgut embedding, will be included in this study. All times, frequencies, and durations of treatment will be eligible for inclusion. Trials will be included if they include any of the following control groups: conventional medication, placebo medication, sham intervention, and no intervention.

#### 2.1.4 Primary outcome

In its most typical form, PTH is clinically similar to other primary headache phenotypes: primarily migraine-like and tension-type headaches, followed by cluster-like, cervicogenic, and other unclassified headache subtypes.

The main measurement of acupuncture efficacy will be evaluated using the Verbal Rating Scale.

#### 2.1.5 Secondary outcomes

The secondary outcomes are the Faces Pain Scale-Revised, Visual Analog Scale, and Numerical Rating Scale.

### 2.2 Search methods

#### 2.2.1 Electronic search strategy

The following electronic databases will be searched from their inception to February 2022: PubMed, Web of Science, Embase, PsycINFO, Chinese databases such as Chinese Biomedicine Literature (CBM), Chinese Medical Current Content (CMCC), Chinese Scientific Journal Database (VIP), WanFang Database, and China National Knowledge Infrastructure (CNKI). No language restrictions will be applied to the search strategy. To ensure an adequate search, we will search for titles, abstracts, and keywords using Medical Subject Headings (MeSH) terms in combination with free text terms. A detailed example of the PubMed search strategy is shown in Table 1.

**Table 1:** PubMed search strategy.

NO.	Search items
#1	Traumatic Brain Injuries
#2	Traumatic Brain Injury
#3	Trauma, Brain
#4	Brain Trauma
#5	Brain Traumas
#6	Brain injury
#7	Brain laceration
#8	Brain concussion
#9	Head injury
#10	Head concussion
#11	Cerebral concussion
#12	TBI
#13	TBIs
#14	Craniocerebral trauma
#15	Encephalopathy, Traumatic
#16	Encephalopathies, Traumatic
#17	Traumatic Encephalopathies
#18	Traumatic Encephalopathy
#19	#1 OR #2 OR #3 OR #4 OR #5 OR #6 OR #7 OR #8 OR #9 OR #10 OR #11 OR #12 OR #13 OR #14 OR #15 OR #16 OR #17 OR #18
#20	Headache^∗^
#21	Post Traumatic Headache
#22	Post-Traumatic Headaches
#23	Cervicogenic Headache
#24	Cervicogenic Headaches
#25	Headache, Cervicogenic
#26	Headaches, Cervicogenic
#27	PTH
#28	#20 OR #21 OR #22 OR #23 OR #24 OR #25 OR #26 OR #27
#29	Acupuncture
#30	Acupoints
#31	Acupunct^∗^
#32	Needling
#33	Electroacupuncture
#34	Pharmacoacupuncture
#35	Moxibustion
#36	Acupressure
#37	Acupotomy
#38	Acupotomies
#39	Elongated needle
#40	Acupoint injection
#41	Auricular needle
#42	Scalp needle
#43	Fire needling
#44	Intradermal needling
#45	Bloodletting therapy
#46	Acupoint catgut embedding
#47	Catgut embedding
#48	Cupping
#49	Meridian
#50	#29 OR #30 OR #31 OR #32 OR #33 OR #34 OR #35 OR #36 OR #37 OR #38 OR #39 OR #40 OR #41 OR #42 OR #43 OR #44 OR #45 OR #46 OR #47 OR #48 OR #49
#51	#19 AND #28 AND #50

∗ Represents 1 or more characters of all characters.

### 2.3 Data collection and analysis

#### 2.3.1 Study inclusion

**Figure 1:**
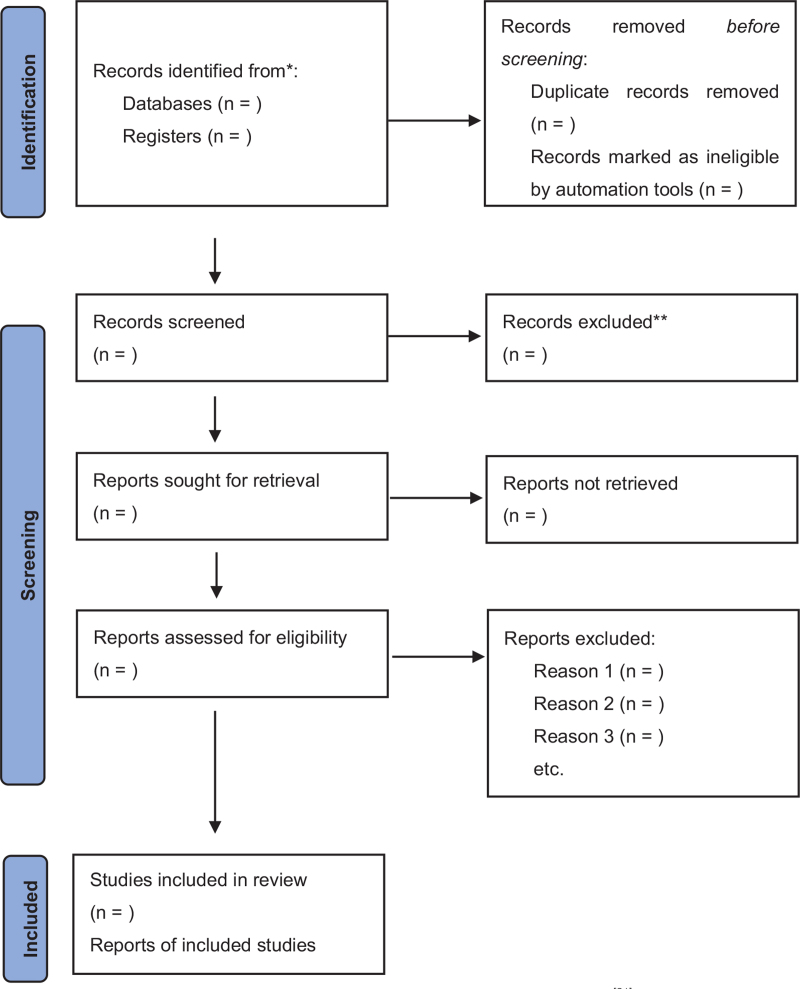
PRISMA 2020 flow diagram for new systematic review.^[21]^

Two researchers will independently screen the literature according to the inclusion and exclusion criteria. They will preliminarily screen the retrieved literature by reading titles and abstracts, crosschecking, and resolving differences through consultation. After preliminary screening, the researchers read the full text carefully and extract the required information. If differences still exist, inclusion is decided in discussion with a third researcher. Eligibility criteria are as follows: The patients who are determined to have a history of TBI. PTH is defined as the onset of headache within 1 week of head trauma or injury or within 1 week after an unconscious patient regains sensation and is able to report pain. The diagnostic criteria of traditional Chinese medicine and western medicine for headache were met. Those who had used other therapies with poor results. Subjects who voluntarily agreed to join the project and signed an informed consent form. Age >18 years. The reasons for the elimination of all excluded studies have been stated. Details of the study selection process are presented in Figure 1.

#### 2.3.2 Data extraction and management

We will build a spreadsheet using Excel to extract the required content, which will then be filled out by 2 reviewers independently. Information will be extracted from eligible articles using standardized data in the following form: the first author, country, publication year, study design, sex, age, randomization method, intervention, sample size, baseline, course of treatment, outcome, adverse events, and follow-up, etc. Two reviewers will then check all extracted data for accuracy and consistency. Disagreements will be resolved through group discussion or consultation with a third reviewer. The trial authors will be contacted for more details and validation if there are any or unclear questions.

#### 2.3.3 Quality assessment and bias risk assessment

The quality of RCT studies will be assessed using the risk of bias assessment tool from the Cochrane Handbook for Systematic Reviews of Interventions, and the quality of cohort and case-control studies will be assessed using the Newcastle–Ottawa Scale. Two reviewers (Yang Yang and Shaowen Hu) will independently extract the data and assess the quality of each study. Disagreements will be resolved by discussion with a third researcher.

#### 2.3.4 Dealing with missing data

If any data from the included trial is insufficiently informative, we will attempt to contact the first or corresponding author to request sufficient information and details. However, if the authors are absent or cannot provide sufficient information, we will conduct a group discussion and analysis based on the available information. In addition, the potential impact of missing data is reported.

#### 2.3.5 Strategy of data synthesis

The meta-analysis will be performed using RevMan 5.3 software. Statistical significance is set at *P* < .05. Fixed-effect (I^2^ < 50%) or random-effect (I^2^ ≥ 50%) models are selected depending on the level of heterogeneity of the included studies. At the same time, sensitivity and subgroup analyses are conducted to determine the possible causes of heterogeneity. However, after sensitivity and subgroup analyses, if the data still had significant heterogeneity (I^2^ > 75%), the meta-analysis would not be accepted.

#### 2.3.6 Subgroup analysis

When heterogeneity exists, subgroup analyses will also be conducted to determine associations between relevant study characteristics such as sex, study quality, sample size, age, acupuncture type and duration, headache severity at baseline, severity of impairment in history of TBI disease, time after injury, psychiatric disorders, and risk of bias.

#### 2.3.7 Sensitivity analysis

A sensitivity analysis will be conducted if there are studies of low quality after conducting a quality assessment of the included researchers. In addition, a sensitivity analysis is performed when there is significant heterogeneity between studies.

## 3 Discussion

PTH after TBI can be caused by trauma to the scalp or skull, mechanical stimulation such as local nerve compression and traction, or by secondary posttraumatic subarachnoid hemorrhage, intracranial hematoma, degeneration and necrosis of brain tissue, chronic hydrocephalus, etc.^[6–8]^ Most of the pain after the recovery period of TBI is related to muscle tension headache, which clinically manifests as dull pain or long-term vague pain.^[9,10]^ The manifestations and recovery of headaches in patients vary, and some of them persist for a long time, seriously affecting the quality of life and even neuropsychological or physiological changes, resulting in mental disorders such as neurasthenia.^[11,12]^ Therefore, early and effective treatment is extremely important. Western medicine can help improve headache symptoms to a certain extent. Simultaneously, the adverse effects of drugs and long-term medication can cause greater psychological stress in patients and serious disturbances in sleep quality.^[13–15]^ Therefore, it is necessary to identify safe and effective treatments that have minimal adverse effects.

Acupuncture and moxibustion are commonly used in Chinese medicine. Many clinical studies have shown that acupuncture can not only help improve headache symptoms, but also has a certain effect on the prevention of the disease and improves quality of life.^[16–20]^ When performing acupuncture treatment, it is necessary to clarify the cause of the headache according to traditional Chinese medicine theory, and the acupuncture points selected are different. We found that there has been no relevant systematic review and meta-analysis reported on this topic in recent years, and this research evaluates the efficacy of acupuncture in the treatment of PTH after TBI by integrating the most comprehensive clinical evidence available. First, we will make every effort to ensure the broadest possible search, including published works in all relevant databases, as well as unpublished works. Second, we will assess the quality of the evidence supporting the effectiveness of the intervention. It is hoped that the results of this systematic review will provide recommendations for clinicians for the clinical management of patients with PTH after TBI.

## Author contributions

**Conceptualization:** Xi Wen, Nenggui Xu.

**Data curation:** Xi Wen, Yang Yang, Tong Liu, Xiaoyin Wang.

**Formal analysis:** Yue Liu, Fangyi Lu, Chanzhen Yu.

**Methodology:** Yunhai Li, Tong Liu, Xiaoyin Wang.

**Resources:** Xiaoyin Wang.

**Software:** Tong Liu.

**Supervision:** Nenggui Xu.

**Writing – original draft:** Xi Wen, Yang Yang.

**Writing – review & editing:** Nenggui Xu.
